# A New Hierarchical Temporal Memory Algorithm Based on Activation Intensity

**DOI:** 10.1155/2022/6072316

**Published:** 2022-01-24

**Authors:** Dejiao Niu, Le Yang, Tao Cai, Lei Li, Xudong Wu, Zhidong Wang

**Affiliations:** Department of Computer Science and Telecommunication Engineer, Jiangsu University, Zhenjiang 212013, China

## Abstract

As a human-cortex-inspired computing model, hierarchical temporal memory (HTM) has shown great promise in sequence learning and has been applied to various time-series applications. HTM uses the combination of columns and neurons to learn the temporal patterns within the sequence. However, the conventional HTM model compacts the input into two naive column states—active and nonactive, and uses a fixed learning strategy. This simplicity limits the representation capability of HTM and ignores the impacts of active columns on learning the temporal context. To address these issues, we propose a new HTM algorithm based on activation intensity. By introducing the column activation intensity, more useful and fine-grained information from the input is retained for sequence learning. Furthermore, a self-adaptive nonlinear learning strategy is proposed where the synaptic connections are dynamically adjusted according to the activation intensity of columns. Extensive experiments are carried out on two real-world time-series datasets. Compared to the conventional HTM and LSTM model, our method achieved higher accuracy and less time overhead.

## 1. Introduction

Hierarchical temporal memory [1] is a machine learning algorithm that simulates the structure and biological functionality of the neocortex and is particularly suitable for sequence learning and prediction. It not only advances our understanding of how the brain may solve the sequence learning problems but also has been applied to various practical implications, such as anomaly detection [2], discrete [3] and continuous sequence modelling [4], face classification [5], handwritten digits recognition [6], and sequence prediction [7].

Compared to the existing machine learning algorithms, HTM exhibits properties that are closer to the working of the human neocortex. It is an emerging brain-inspired cognitive and computing model derived from the discoveries of neuroscience and brain science [8]. Classic artificial intelligence (AI) and artificial neural networks (ANNs) generally are designed to solve specific types of problems rather than proposing a general theory of intelligence. In contrast, HTM tends to seek solutions that are universal in that they apply to every sensory modality. Therefore, HTM is a theoretical framework for both biological and machine intelligence [9].

As the name implies, HTM is fundamentally a memory-based system. It is trained on lots of time-varying data and relies on storing a large set of patterns and sequences. The implementation of HTM mainly involves two modules: spatial pooler (SP) and temporal memory (TM). The SP algorithm converts the input data into sparse distributed representation (SDR) with fixed sparsity, whereas the TM algorithm learns sequence and makes context-sensitive predictions.

The HTM model consists of regions or levels arranged in a hierarchical form. Each region is composed of neurons known as cells, and multiple cells are aligned vertically to form a column. The column and cell are the fundamental building blocks in HTM. Each cell has a proximal dendritic segment and multiple distal dendritic segments. Each segment contains sets of synapses that are characterized by a scalar permanence value. All cells on the same column share the proximal segment, whereas the cell on distal segments receives lateral inputs from nearby cells.

The HTM column has two states: active and nonactive, and the cell has three states: active, predictive, and nonactive. Each synapse on the shared segment connects to an input data bit, and multiple columns become active once the input is read in; that is, the input data are represented as a set of active columns. Then, some cells (predictive cells at the previous time step) on the active columns will become active. All synapses on the distal segments of active cells are checked, if the presynaptic cell of the synapse is currently activated, the postsynaptic cell will become predictive now. Thus, the input data under a certain context are represented by a set of active cells. Finally, the set of predictive cells is decoded to form the prediction results of the next time step.

Typically, SDR is the key characteristics of the HTM model. As the SDR of SP, the active columns encode the spatial pattern of the input and are sent to TM. However, using the representation of active columns may lose some important information of input bits. The state of *active* merely indicates the column is closely related to the current input. More detailed information, such as the closeness of the connection and its effect on the subsequent TM learning, is ignored. Moreover, the conventional HTM, both the SP algorithm and TP algorithm, uses an online unsupervised Hebbian-style learning rule [10] to adjust the synaptic connection. The permanence value of synapse is increased or decreased by a fixed learning rate, without considering the impact of various columns and cells but imposing the same reward or punishment indiscriminately. In general, these limitations make the HTM model less flexible, weaken its internal representation ability, and subsequently impede its performance.

To address the above problems, we propose a new HTM algorithm based on activation intensity with the aim to extend the representation capability and improve the HTM learning. The activation intensity is introduced to describe the degree to which the column is associated with the input pattern. Compared to the conventional column state, activation intensity enables a fine-grained abstraction on the input and provides more detailed information of synaptic connection. Furthermore, an improved self-adaptive learning strategy that exploits the column activation intensity is proposed for more effective sequence learning.

The main contributions of our work are as follows:

1. By introducing the column activation intensity, a new HTM is proposed to improve the conventional HTM performance on sequence learning.
2. To improve the representation capability of HTM, the activation intensity and the column state are combined to learn the spatial patterns of the input data. With the activation intensity, more useful information is maintained on the column and provided for more accurate context learning.
3. To make the training perfectly adapt to various input contexts, a column-sensitive learning strategy is proposed for synaptic permanence adjustment. Synaptic connections are adaptively adjusted according to the activation intensity of columns where they locate. Thus, the cells are able to learn more dynamically and discriminatively.
4. The proposed HTM system is implemented on the NuPIC open-source platform. The experimental results on two time-series datasets, NAB and NYC-Taxi, show that the proposed HTM is able to improve the prediction accuracy and significantly reduce the training time overhead compared with the conventional HTM and the LSTM network.

## 2. Related Works

### 2.1. HTM

In recent years, a lot of research efforts have been devoted to HTM. In [11], novel HTM approaches were proposed to encode coincidence-group membership (fuzzy grouping) and to derive temporal groups (maxstab temporal clustering). Systematic experiments on three line-drawing datasets have been carried out to better understand HTM peculiarities and verified that the proposed approaches have higher accuracy than other traditional pattern recognition methods. In [12], the prediction accuracy of HTM was compared with several sequence learning algorithms including LSTM, the extreme learning machine [13], the autoregressive integrated moving average [14], the echo state network [15], and the time-delayed neural network [16] on both artificial and real-world data. The results revealed that HTM achieves a prediction accuracy that is comparable to or better than the other state-of-the-art algorithms.

To avoid setting the parameters of HTM, Suzugamine et al. [17] proposed a self-structured HTM that dynamically adjusts the number of columns and cells according to the input data. The results on time-series test input and real-world power consumption show the proposed method achieves a higher prediction accuracy than LSTM and HTM. In [18], a decoder of internal prediction representation in HTM was proposed. The proposed method decodes the representation by utilizing the relationship between the input data bits and the columns built during the encoding process, and thus avoids the additional learning process and achieves smaller prediction error.

To detect machine failures preemptively and reduce production costs, Malawade et al. [19] proposed a method of performing online, real-time anomaly detection for predictive maintenance using HTM and compared with KNN-CAD, Windowed Gaussian, and other detectors in the Numenta Anomaly Benchmark [20]. The proposed method verified the robustness and adaptability of HTM. In [21], Jakob et al. proposed HTMRL, a bio-feasible reinforcement learning algorithm based on HTM, which supports nonstationary environments. HTMRL performs well on a 10-armed bandit task and spends less time to adapt to the bandit suddenly shuffling its arms. Zyarah and Kudithipudi [22] proposed a comprehensive neuromemristive crossbar architecture for the spatial pooler and the SDR classifier structure for mobile devices and energy-constrained platforms. The proposed design has high-speed calculation, low power consumption, and reconfigurability, which verifies that the HTM architecture can accurately identify images even in the presence of noise.

Osegi [23] applied HTM into the task of short-term load forecasting using spatial pooler and a temporal aggregator, which transform SDRs into a sequential bivariate representation and makes temporal classifications from the SDRs. They verified that HTM has stronger noise resistance and can outperform most existing artificial intelligence neural technologies in short-term load forecasting tasks. Hawkins et al. [24] proposed a framework based on location information to describe the function of the new cerebral cortex, which verified that HTM can also show higher performance and advantages in the face of multiprediction tasks.

The above research studies show that HTM has better performance than traditional machine learning algorithms in many fields. However, few works consider the limitations of internal representation of HTM learning components and their affects to the performance of HTM. In this work, we aim at improving the representation ability of HTM and exploring a more flexible learning method to increase its performance.

### 2.2. Hierarchical Temporal Memory Network

**Figure 1:**
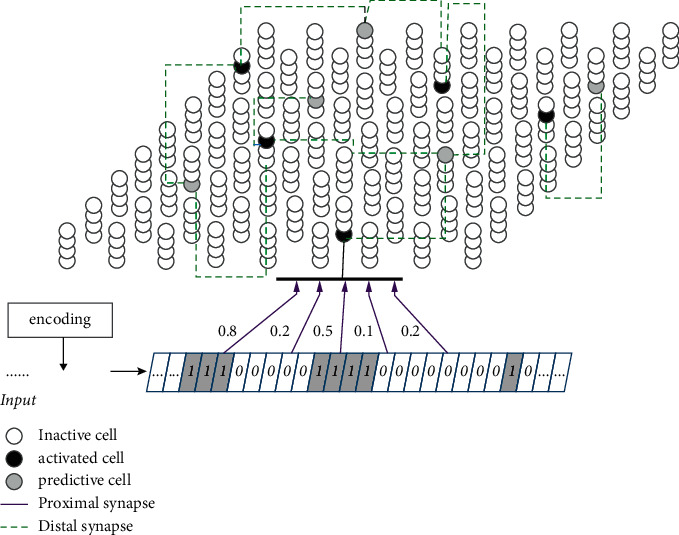
A structure of the HTM region (single level). The region consists of the column of vertically stacked cells (best seen in colour).

A single-level HTM network structure is illustrated in Figure 1, which consists of one region. Each region is composed of some columns *colj* (*j* = 1, 2,…, *n*_col_) that have multiple cells *ce*_*j*_^*i*^ (*i* = 1, 2,…, *n*_ce_) on it (here *n*_col_ = 81, *n*_ce_ = 4). The cells are the basic learning opponents that are used to emulate the functionality of the pyramidal neurons. Each HTM column shares a proximal dendrite segment (bold black line), and each cell has a dozen or two distal dendrite segments (not shown). The proximal dendrite segment receives feedforward input, and the distal dendrite segments receive lateral input from nearby cells through the synapses on each segment. The proximal synapses and the distal synapses are shown in purple and green in Figure 1, respectively.

The HTM model mainly includes two modules: spatial pooler (SP) and temporal memory (TM). Each of the modules is briefly described as follows.

### 2.3. HTM Spatial Pooler

The main function of SP in HTM is learning to recognize certain patterns of the input data such that for different inputs that possess similar characteristics, particular attributes in the output are activated [25]. In other words, SP forms an internal representation of the input data using SDR, which is characterized by a set of active columns in the HTM model.

The SP algorithm contains three phases: initialization, column activation, and proximal synaptic permanence learning. Initialization determines the main parameters of the HTM model and builds connections between the column and the input space through potential synapses on proximal dendritic segment. Each column col_*j*_ is connected to the part of the input space, which refers to as the receptive field (*D*_*j*_). *D*_*j*_ is defined either locally or globally. In the local receptive field, the *j-*th column synapses will be connected to a predefined region centered at *x*_*j*_^*c*^ with a range *γ*, whereas in the case of the global receptive field, the synapses can be connected anywhere in the input space. The following equation represents the potential connections PI (*j*) between the input *x* and *j-*th column:

(1) $$\begin{array}{c} PI\left(j\right)=\left\{z\;|\;\sigma \left(x_{z};x_{j}^{c},\gamma \right)\text{and}\left(p_{jz}∼U\left(0,1\right)\right)\right\}, \end{array}$$

where *σ*(*x*_*z*_; *x*_*j*_^*c*^, *γ*) = 1, ∀*x*_*z*_ ∈ (*x*_*j*_^*c*^, *γ*), and *p*_*jz*_∼*U* (0, 1) represents the synapse permanence of the *j-*th column to the *z* bit of the input data and is selected randomly from the uniform distribution *U* having range [0, 1]. Those potential synapses are considered to be connected if their permanence value exceeds the threshold *θ*_*c*_.

The column activation process selects a set of active columns (also called winning columns) to represent the feedforward input. The overlap value determines the activation of SP columns for a given input pattern *x* and is calculated by counting its active synapses that associate with the active bits of input data as shown in the following equation:

(2) $$\begin{array}{c} o_{j}=\beta_{j}\sum_{z}B_{jz}x_{z}, \end{array}$$

where *β*_*j*_ is the boosting factor that determines the excitability of each SP column, which is modified during the training of SP; and *B* is an indicator matrix with *B*_*jz*_ = 1 if *p*_*jz*_ is greater than the connection threshold *θ*_*c*_, while *B*_*jz*_ = 0 otherwise.

In the inhibition, the columns within the inhibition radius *ξ* and the overlap value in the top *k* are activated and represent the feedforward input, while other adjacent columns will be inhibited, as given by

(3) $$\begin{array}{c} a\_col^{t}=k\_\max\left(o_{j},\xi \right). \end{array}$$

We denote *k_*max( ) as a function that implements *k*-winner-take-all on the permanence value.

In the learning phase, the synaptic permanence values of the active columns are updated using the Hebbian learning rule [10]. The rule implies that the synapses connected to the active input bits must be strengthened, increasing their permanence by *ρ*^*+*^, whereas the synapses connected to the inactive bits must be weakened, decreasing their permanence by *ρ*^−^.

### 2.4. HTM Temporal Memory

The HTM TM learns sequence and forms a representation in the context of previous inputs based on the active columns, and then makes predictions for the future input. The cells of the active columns are involved in this process. The TM algorithm mainly includes three phases: determining the active cells of the columns, distal synaptic permanence learning, and setting the cells in the predictive state.

First, TM will activate some cells on the active columns based on historical information. The calculation of the active state is shown as follows:

(4) $$\begin{array}{c} a_{ji}^{t}=\left\{\begin{array}{cc} 1, & if\;j\in a\_col^{t}\;and\;\pi_{ji}^{t-1}=1\\ 1, & if\;j\in a\_col^{t}\;and\;\sum_{i}\pi_{ji}^{t-1}=0\\ 0, & \text{otherwise} \end{array}\right), \end{array}$$

where *π*_*ji*_^*t*−1^ denotes the predictive cell at time *t*-1 on *j-*th column *i-*th cell. For active columns that contain predictive cells of *t* − 1, those cells are set to be active of the current time step. Meanwhile, for active columns that do not have predictive cells of *t* − 1, all cells on those columns will be activated. Unlike the active columns in SP that characterize the current input, TM further represents the different contexts by the active cells on these columns, that is, learning the temporal dependencies within the data sequence. Even for the same input under different contexts, the internal representation of TM is quite distinct.

Learning in TM and SP is similar, where the Hebbian rule is used to adjust the permanence values of the distal synapses. The learning cells that connected to the active cells of the previous time step have their dendritic segments positively reinforced by a larger value *ρ*+, whereas the cells connected to the nonactive cells have the segment negatively reinforced by a small value *ρ*^−^:

(5) $$\begin{array}{c} \Delta {\text{P}}_{ij}^{d}=\rho^{+}{\hat{\text{P}}}_{ij}^{d}\circ A^{t-1}-\rho^{-}{\hat{\text{P}}}_{ij}^{d}\circ \left(1-A^{t-1}\right). \end{array}$$

*P*_*ij*_^*d*^ is an *M* × *N* matrix denoting the permanence of the *d-*th segment of the *i-*th cell in the *j-*th column. *A*^*t*^ is an *M* × *N* binary matrix, where *a*_*ij*_^*t*^ is the activation state of the *i-*th cell in the *j-*th column. ${\hat{P}}_{ij}^{d}$ denotes a binary matrix containing only the positive entries in *P*_*ij*_^*d*^, that is,

(6) $$\begin{array}{c} {\hat{\text{P}}}_{ij}^{d}=\left\{\begin{array}{cc} 1 & if\;{\text{P}}_{ij}^{d}>0\\ 0 & \text{otherwise} \end{array}\right). \end{array}$$

Finally, TM predicts the possible output in the next time step based on the active cells determined in the first phase. All distal segments are examined where the segments in the active state turn their cell in the predictive state unless the cell is already activated by the feedforward input. Whether the distal segment is active depends on the number of active synapse connected to the active cell. Once the number exceeds the threshold *θ*_*s*_, the segment will be in the active mode. Thus, the predictive state of a cell at time step *t* is given by

(7) $$\begin{array}{c} \pi_{ji}^{t}=\left\{\begin{array}{cc} 1, & if\;\exists_{d}||DS_{ji}^{d}\cdot A^{t}|{|}_{1}>\theta_{s}\\ 0, & \text{otherwise} \end{array}\right), \end{array}$$

where *DS*_*ji*_^*d*^ is the *d-*th distal segment of the *i-*th cell within the *j-*th column, and *A*^*t*^ is the matrix on *a*_*ji*_^*t*^ size of *n*_col_ × *n*_*ce*_.

The predictive cells are the output of TM and will then be decoded into the same format of input using a maximum-likelihood classifier [12].

### 2.5. Problems in Conventional HTM

From the above analysis, we find some potential problems of the conventional HTM.

First, the HTM SP uses the set of active columns as output to form a sparse distributed representation of the input data. According to the SP algorithm, HTM picks the top *k* columns with the highest overlap value and places them into the active state. However, this simple processing method ignores the inherent difference within the active columns. In fact, the representation capabilities differ between various active columns.

**Figure 2:**
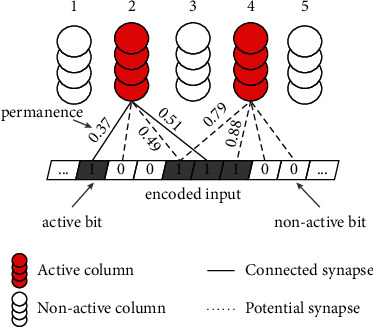
Two active columns in HTM.

Figure 2 shows the proximal dendritic segment of three columns. The red columns are active because their overlap values exceed the threshold *θ*_*o*_ (*θ*_*o*_ = 2) and are among the k-highest ones, yet the synaptic connections on the active columns (column 2 and column 4) differ substantially in terms of the permanence value and the numbers of connected synapses. Column 2 has three connected synapses with permanence 0.37, 0.49, and 0.51, whereas column 4 has two connected synapses with permanences of 0.79 and 0.88. Although the two columns are both active, their synaptic connections with the input are not exactly the same. In this case, column 4 seems more closely related to the current input than column 2. Unfortunately, the state of “active” is inadequate to reflect these subtle distinctions. When input data arrive, SP should not only select the columns that are strongly associated with the specific input patterns, but also keep the detailed information about these associations and use them to guide the learning.

Second, when TM learns the sequence, the permanence value between distal synaptic connections is adjusted by a fixed learning rate. All learning cells apply the same updating *ρ*^*+*^ and *ρ*^−^ for strengthening or weakening without considering the differences of these cells, which may lead to a poor performance and low flexibility. Intuitively, the synapses of the active cells that locate on the strongly associated columns should update more significantly and quickly than those connected to weakly related columns.

To address the above problems in conventional HTM, we propose a novel HTM learning algorithm based on activation intensity. Besides the column state, the activation intensity, which describes the strength of the synaptic connection, is introduced and leveraged for input representation. The column activation intensity is able to enhance the representation ability of SDR in SP. Furthermore, we explore a self-adaptive training method for TM based on the activation intensity, where the synapses of learning cells are adjusted in a column-sensitive manner. The details of the proposed method are given as follows.

## 3. Hierarchical Temporal Memory Based on Activation Intensity

### 3.1. Activation Intensity

We expand the state of column and introduce the activation intensity to further enhance the learning ability of SP. The activation intensity refers to the activation degree under a given input. From Figure 2, we can find active columns 2 and 4 have different numbers of active synapses and their permanence values vary substantially. Combining these factors, the activation intensity is defined as follows:

(8) $$\begin{array}{c} AI_{j}^{t}=sigmoid\left(\sum_{z}B_{jz}x_{z}+\alpha ||pc_{j}^{t}|{|}_{2}\right), \end{array}$$

where *AI*_*j*_^*t*^ denotes the activation intensity of the *j-*th column at time *t*, **pc**_*j*_^*t*^ is the permanence vector of connected synapses on the *j-*th column, and *α* is a coefficient. The sigmoid function is used to keep the intensity value in (0, 1).

The activation intensity includes two parts. The first part represents the number of connected synapses on the *j-*th column connected to the active input bits. This is a term related to the overlap, where the boost factor *β*_*j*_ is removed. For a column, the more valid the synapses are formed, the more useful the information will be transmitted to the column. In Figure 2, the activation intensity of column 2 is higher than that of column 4 because it has more valid synapses than column 4.

The second part in the definition concerns the permanence of these synapses, where L-2 norm is imposed on the permanence vector. For a valid synapse, the greater the permanence value is, the stronger the connection with the input will be. Once a column is tightly connected with a input pattern, the stimuli from the presynapse will be easily arrived in that column. In Figure 2, column 4 has comparatively higher permanence on all valid synapses, while less connections are built. To combine these two factors and balance their contribution, a coefficient *α* is adopted in activation intensity. Finally, the sigmoid function is used to restrict the output to the desired range.

### 3.2. A Self-Adaptive Temporal Memory Learning Algorithm Based on Activation Intensity

Based on the activation intensity, we further improve the TM algorithm and propose an adaptive temporal memory algorithm for sequence learning. The idea behind it is that the obtained active columns and their intensities are exploited to update the synapses on distal dendritic segments.

The proposed TM learning algorithm uses a self-adaptive learning strategy to dynamically adjust the synaptic connections between the cells. Specifically, when the cells on the active columns are updating their permanence, the amount of update is calculated according to the activation intensity of the column. Cells on high-intensity columns learn faster than those on lower-intensity columns, thus having their synaptic permanence updated by a greater extent. On the contrary, cells on the lower-intensity column should take less update. Other synapses on the matching segment of nonactive columns that connects to the active cells of time *t*-1 are weakened by a less extent because the postsynaptic cell of that connection is on the nonactive column. The following equation gives the self-adaptive permanence updating policy:

(9) $$\begin{array}{c} \Delta {\text{P}}_{ij\;d}^{s}=\varphi^{+}\left(AI_{j}^{t},AI_{\text{col}\left(s\right)}^{t-1}\right){\hat{\text{P}}}_{ij}^{d}\circ A^{t-1}-\varphi^{-}\left(AI_{j}^{t},AI_{\text{col}\left(s\right)}^{t-1}\right){\hat{\text{P}}}_{ij}^{d}\circ \left(1-A^{t-1}\right), \end{array}$$

(10) $$\begin{array}{c} \varphi^{+}\left(AI_{j}^{t},AI_{col\left(s\right)}^{t-1}\right)=e^{-\left(AI_{j}^{t}+\omega \right)}+e^{-\left(AI_{col\left(s\right)}^{t-1}+\omega \right)}, \end{array}$$

(11) $$\begin{array}{c} \varphi^{-}\left(AI_{j}^{t},AI_{\text{col}\left(s\right)}^{t-1}\right)=\max\left(0,\;\left(e^{-\left(AI_{j}^{t}+\omega \right)}-e^{-\left(AI_{col\left(s\right)}^{t-1}+\omega \right)}\right)\right), \end{array}$$

where ΔP_*ij*  *d*_^*s*^ represents the adjustment for the *s-*th synapse on the *d-*th segment of the *i-*th cell within the *j-*th column. The increase and decrease in the permanence value are calculated by *φ*^+^(·) and *φ*^−^(·), two nonlinear functions proposed for dynamic permanence adjustment shown in (10) and (11), *AI*_*j*_^*t*^ and *AI*_*col*(*s*)_^*t*−1^ are the activation intensities at time *t* and *t*-1 for the *j-*th column and the column where synapse *s* locates, respectively, and *ω* is a tuning parameter, and we will investigate its sensitivity on the results in the experiment.

With the new learning strategy, the permanence updating is performed in a column-sensitive manner. Unlike the traditional TM learning, which takes a fixed update for all learning cells, the proposed algorithm gives each cell an individual update, adjusting the synaptic connections based on the activation intensity of the column where the cell locates. Different synapses may take different updating rates. Meanwhile, the use of a nonlinear function ensures that the synapse can take different updates at various time steps.  Algorithm 1 summarizes the proposed TM learning algorithm:

## 4. Experiments

### 4.1. Comparison Algorithms

We compare the performance of the time-series prediction of the conventional LSTM, HTM, and the proposed HTM. For simplicity, the HTM based on activation intensity is termed HTM_AI in the following sections. For the conventional LSTM and HTM, we utilize the implementations in DeepLearning4j and NuPIC, respectively.

### 4.2. Experimental Setting

We implemented the proposed HTM network on the NuPIC's open-source HTM framework. To decode prediction values from the output of SDRs of HTM, we consider two classifiers as in [12]: a simple classifier based on SDR overlaps and a maximum-likelihood classifier. For the prediction on SDR representation, we computed the overlap of the predicted cells with the SDRs of all observed elements and selected the one with the highest overlap. For the prediction on continuous scalar value output, we divided the whole range of scalar value into 22 disjoint buckets and used a single-layer feedforward classification network.

Tables 1 and 2 show the main parameters of the proposed model and the baseline method. Besides these parameters, the real-valued input data are transformed into binary data bits **x**^*t*^=(*x*_1_^*t*^,  *x*_2_^*t*^,  ... ,  *x*_*n*_^*t*^) with *n* = 104 bits. The decoding classifier for HTM and HTM_AI uses 128 neurons. For the conventional LSTM, we employed one hidden layer with 32 neurons and mean-squared error was used as the loss function.

### 4.3. Datasets

**Figure 3:**
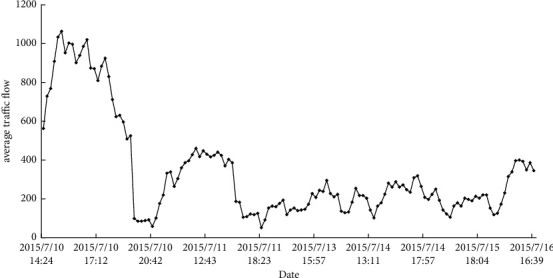
Data distribution of NAB.

Numenta Anomaly Benchmark (NAB) provides a standard, open-source framework for evaluating real-time anomaly detection algorithms on streaming data. It comprises two main components: a scoring system designed for streaming data and a dataset with labelled, real-world time-series data. We chose the vehicle traffic dataset from the Minnesota Department of Transportation for evaluation. Each item is composed of time stamp and numerical data. The data record the average traffic time of all vehicles in each month in the Twin Cities area of Minnesota at ten-minute intervals, with a total of 2500 vehicles.  Figure 3 shows the data distribution.

**Figure 4:**
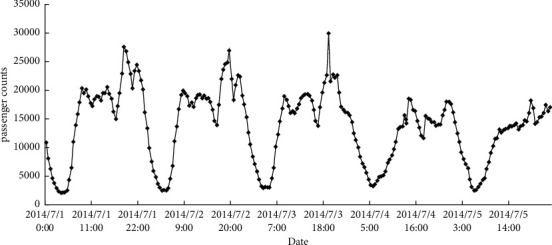
Data distribution of NYC-Taxi.

NYC-Taxi is a public data stream provided by the New York City Transportation Authority. The data are collected in real-world scenarios and contain 10320 continuous data streams. We aggregated the passenger counts in New York City taxi rides at 30-minute intervals and took the taxi passenger prediction task to evaluate the proposed method. Compared with the NAB dataset, the sequence exhibits rich patterns at different timescales (see Figure 4). To compare the performance of our HTM with other sequence learning techniques, we predict taxi passenger demand five steps (2.5 hours) in advance.

### 4.4. Evaluation Metric

As the evaluation metric, we used the prediction accuracy (Accuracy), which measure the overlapping probability between the output SDR at time *t* and the SDR of input at time *t*+1 given by

(12) $$\begin{array}{c} \text{accuracy}=\text{ave}\left(\frac{\text{overl}\left(SDR_{o}^{t},S\;DR_{i}^{t+1}\right)}{\left|SDR_{i}^{t+1}\right|}\right), \end{array}$$

where *SDR*_*o*_^*t*^ and *SDR*_*i*_^*t*+1^ represent the SDR output at *t* and the SDR input at *t* + 1, respectively. The function overl( ) calculates the overlapping ratio of two SDR representations. ave( ) gives the average accuracy on all samples. |·| means the length of SDR vector. In the experiment, we chose 40 active columns to form the SDR. A large SDR overlapping indicates a higher prediction algorithm. All the results are reported on 20 runs of the average values.

Besides accuracy, we also take the root mean square error (RMSE), which measures the difference between the true and predicted values as the other metric. A small prediction error indicates a better prediction algorithm. The *RMSE* is given by

(13) $$\begin{array}{c} \text{RMSE}=\sqrt{\frac{1}{T}\sum_{t=1}^{T}{\left({\bar{y}}^{t}-y^{t}\right)}^{2}}, \end{array}$$

where *y*^*t*^ is the actual value of the observation at time *t*, ${\overset{-}{y}}^{t}$ is the model prediction, and *T* is the total number of predictions.

## 5. Results and Discussion

### 5.1. Prediction Accuracy on NAB

**Figure 5:**
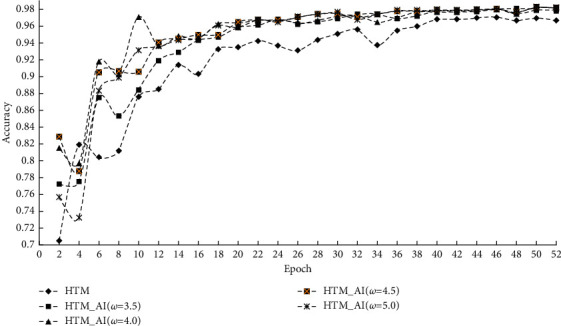
Prediction accuracy on NAB.

First, we illustrate the prediction accuracy of different methods on the NAB dataset.  Figure 5 shows the prediction accuracy under various running epochs. To examine the impact of column activation intensity on the prediction accuracy, we chose four different values, namely *ω* = 3.5, 4.0, 4.5, and 5.0.

**Figure 6:**
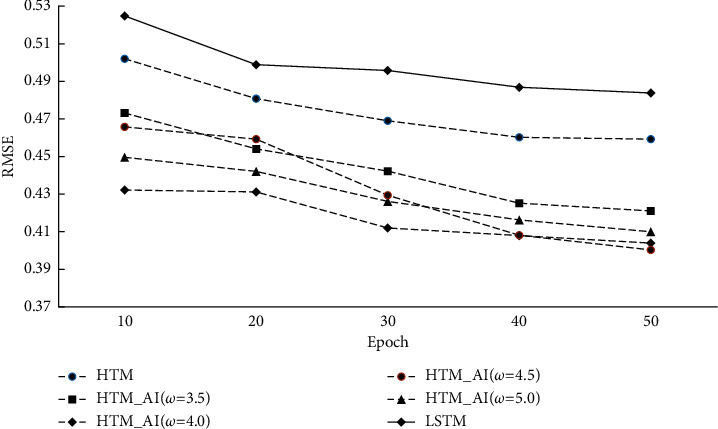
Prediction error (RMSE) on NAB.

Figure 6 gives the RMSE results on conventional HTM, HTM_AI, and LSTM. In order to acquire RMSE, a decoder is used after HTM and HTM_AI implement the TM learning. We also report the performance of the LSTM network where the predicted output value is compared with the true value. For our HTM_AI, different intensity thresholds are chosen for comparison.

From the results, we see that the accuracy and the prediction error vary with different *ω*. However, HTM_AI consistently has a higher accuracy than the conventional HTM except when the epoch is 4 in Figure 5. After that, the accuracy of HTM_AI increases continuously. When the epoch exceeds 30, the prediction accuracy tends to be stable around 0.98. For different values of *ω*, the accuracy changes greatly in the early stage of HTM_AI training. However, as a general tendency, the accuracy under different *ω* finally inclines to be very similar when the training converges.

From Figure 5, we can find the accuracy of conventional HTM is lower than the proposed method, even if the training is stable after 40 epochs. Because the accuracy in Figure 5 is calculated based on the SDRs, the decoder classifier can't affect the results. For the prediction error, Figure 6 shows HTM_AI has a lower RMSE value under the same running epochs. Compared with the LSTM network, HTM_AI is able to reduce RMSE by up to 8% in the case of epoch = 50 and *ω* = 4.5. Even if the decoding classifier is involved in calculating the scalar output, HTM_AI still shows superior performance than LSTM. HTM_AI also performs better than the conventional HTM under various parameter settings.

**Figure 7:**
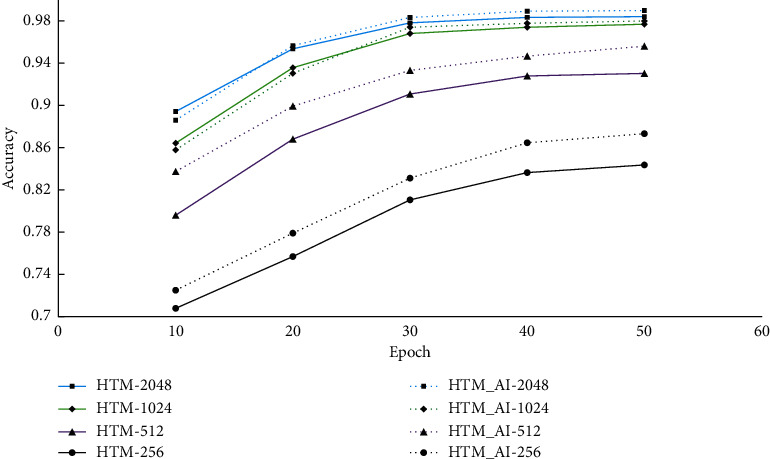
Prediction accuracy with different numbers of columns on NAB.

Next, we also tested the impact of model parameters on the prediction performance. We give the results with different numbers of columns and neurons. From the prediction accuracy shown in Figure 7, we find the accuracy is proportional with the column number, more columns leading to a higher accuracy. When we adopted 2048 columns, both the conventional HTM and the proposed HTM show the highest accuracy and the performance maintains stable when epoch exceeds 40. So, for the NAB dataset, 2048 or 1024 columns seems to be a better choice.

As for the number of neurons, we take 8, 16, 24, 32 and fix the epoch to 50. All results are tested 20 times, and the average value is finally reported on Table 3.

These results reveal that the HTM with activation intensity can improve prediction performance on irregular time series. More information obtained from SP can help temporal dependency learning in TM and thus leads to an improved model performance.

### 5.2. Time Overhead on NAB

Here, we discuss the time overhead on training HTM and HTM_AI. Because LSTM usually uses an offline training, whereas HTM is trained by an online manner, we only report the time overhead on HTM_AI and HTM. In this experiment, the expected accuracy is set to 80%, 85%, 90%, and 95%, respectively. The focus is to check how the time overhead changes in order to meet the desired accuracy.

**Figure 8:**
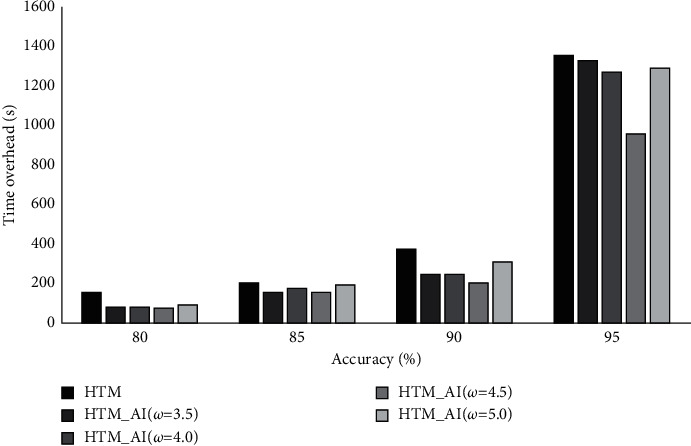
Training time overhead comparison on NAB.

Figure 8 shows the average time cost. From the results, we can see that the proposed HTM_AI needs comparatively less time to reach the desired accuracy. The gap between HTM and HTM_AI (*ω* = 5.0) is not obvious. However, for HTM_AI (*ω* = 4.5), the training time is much lower than HTM. For the NAB dataset, the least time is acquired when *ω* is set to 4.5, which is consistent with the result on accuracy evaluation. The figure also illustrates that when the accuracy increases from 0.9 to 0.95, the rising of training time is the highest, growing nearly 3.7 times. To reach the accuracy of 0.95, HTM_AI can reduce 29% of training time compared to the conventional HTM. When the accuracy increases, HTM_AI shows obvious advantage over its counterpart on time overhead.

These results reveal that our HTM can reduce the training time and make the training reach the desired accuracy much faster. The possible reason is that the TM algorithm can utilize the activation intensity to speed up building connections between the active cells. Columns that are closely related to the input pattern will have more permanence changes on their synapses. The results verify this dynamic learning strategy makes HTM training more efficient.

### 5.3. Prediction Accuracy on NYC-Taxi

**Figure 9:**
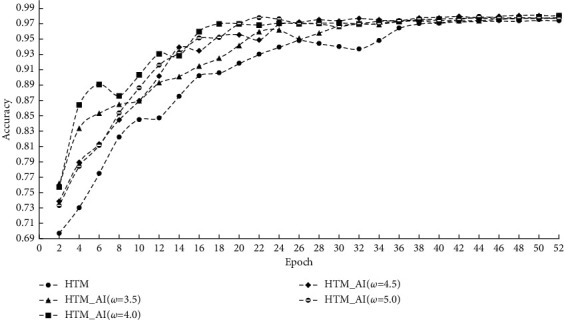
Prediction accuracy on NYC-Taxi.

Then, we verify the effects of the proposed HTM_AI on the NYC-Taxi dataset. The dataset exhibits more cyclical patterns compared to NAB, and the cycle may change from just a few hours to a day, a week, or even a quarter, making the prediction more challenging. We reported the prediction accuracy every two epochs and run totally 52 epochs. The results are shown in Figure 9.

The parameters remain the same as the previous section. From the results, we can find that at the beginning, HTM shows the lowest accuracy, whereas the proposed HTM_AI already has higher accuracy. As the training continues, both models tend to raise their accuracy. After the training is more than 40 epochs, the gap between the two models gradually decreases. On this dataset, the different parameter values only impact the early stage of training and the optimal value varies as training goes on. However, in all cases the proposed HTM_AI shows 0.8%∼1.2% higher accuracy than the conventional HTM.

The results reveal that the HTM with activation intensity is able to improve the prediction accuracy. Even at the initial stage, it has achieved superior performance than the conventional HTM, which indicates the activation intensity help HTM learn input patterns more effectively. Due to the complex cyclic property of the input data, the convergence is relatively slowly than that of the NAB dataset.

**Figure 10:**
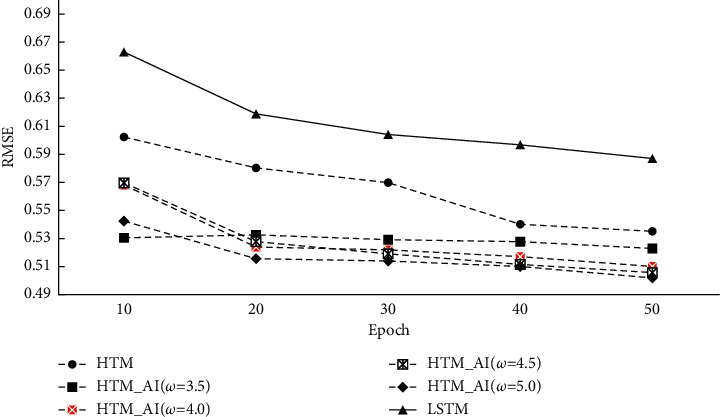
Prediction error (RMSE) on NYC-Taxi.

Figure 10 shows the results of prediction error. RMSE curves are very similar to the results of the NAB dataset. The difference is that the lowest error obtains when *ω* is 5.0 instead of 4.5 in NAB. Compared to LSTM, the prediction error RMSE reduces by 10%∼14.1%. The results indicate that on different datasets, the proposed HTM always outperforms the conventional HTM and LSTM.

**Figure 11:**
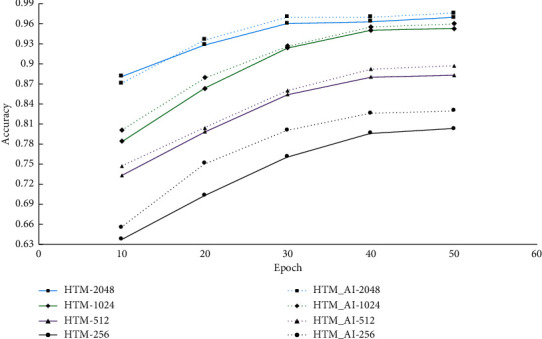
Prediction accuracy with different numbers of columns on NYC-Taxi.

Then, we also investigate the impact of model size on the performance. As in the NAB dataset, we set the number of columns and neurons to 256, 512, 1024, and 2048 and 8, 16, 24, and 32, respectively. The prediction accuracy is shown in Figure 11 and Table 4.

We find both HTM and HTM_AI achieve the highest accuracy when the number of columns is 2048, which is consistent with that of NAB. This result also verifies that HTM is different from popular neural networks, such as RNNs, of which the optimal model size is problem-dependent. HTM usually adopts a fixed column number, and 2048 is regarded as a good option by all prior research literature studies [7]. From Table 4, we observe that the prediction accuracy remains nearly the same with different numbers of neurons on a column. Compared with the column number, the accuracy is not significantly affected by the number of neurons.

### 5.4. Time Overhead on NYC-Taxi

**Figure 12:**
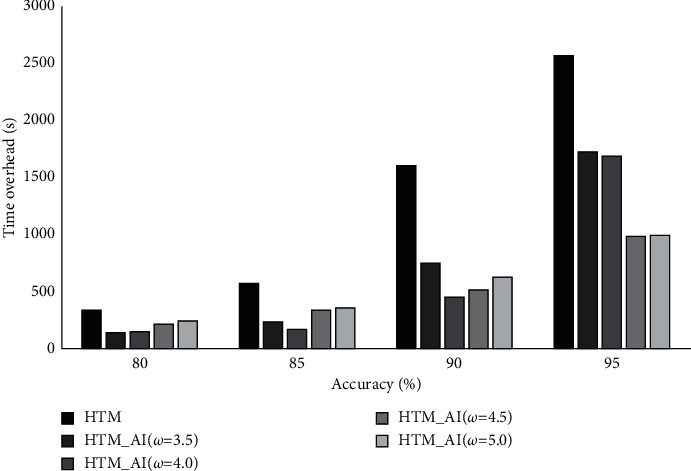
Training time overhead comparison on NYC-Taxi.

We also report the time overhead on the NYC-Taxi dataset. The desired accuracy is set to 0.8, 0.85, 0.9, and 0.95, respectively.  Figure 12 shows the time for training the conventional HTM and HTM_AI with different parameters.

Unlike the NAB dataset, NYC-Taxi has more samples and involves rich patters in terms of cyclic temporal dependency, so the training takes comparatively longer time than NAB. From the results in Figure 12, it can be seen that on reaching the same accuracy, HTM_AI needs about 30%–61% less time than HTM. The results are consistent with the accuracy in Figure 9. With the activation intensity, HTM_AI can achieve higher accuracy since the training starts. For the NYC-Taxi dataset, a larger parameter value seems benefit for HTM_AI training. A possible reason may lie in complex pattern that needs more representative columns and stronger synaptic connections to learn.

### 5.5. Long-Term Prediction Results

**Figure 13:**
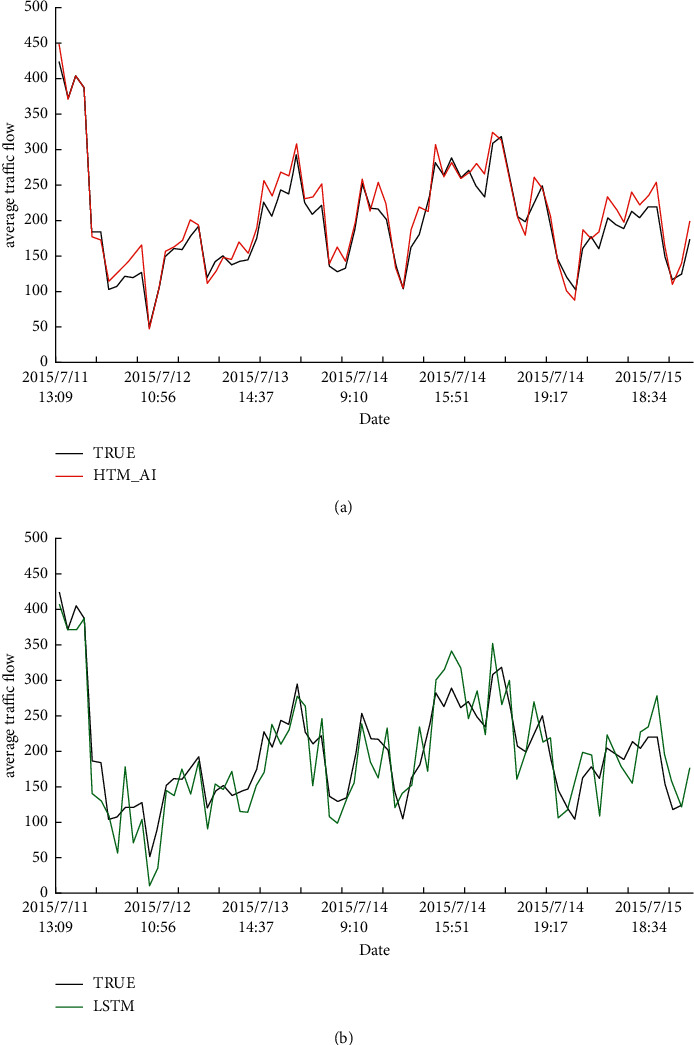
Predicted average traffic flow. True data are shown in black. (a) The prediction results using HTM_AI, shown in red. (b) The prediction result using LSTM, shown in green.

**Figure 14:**
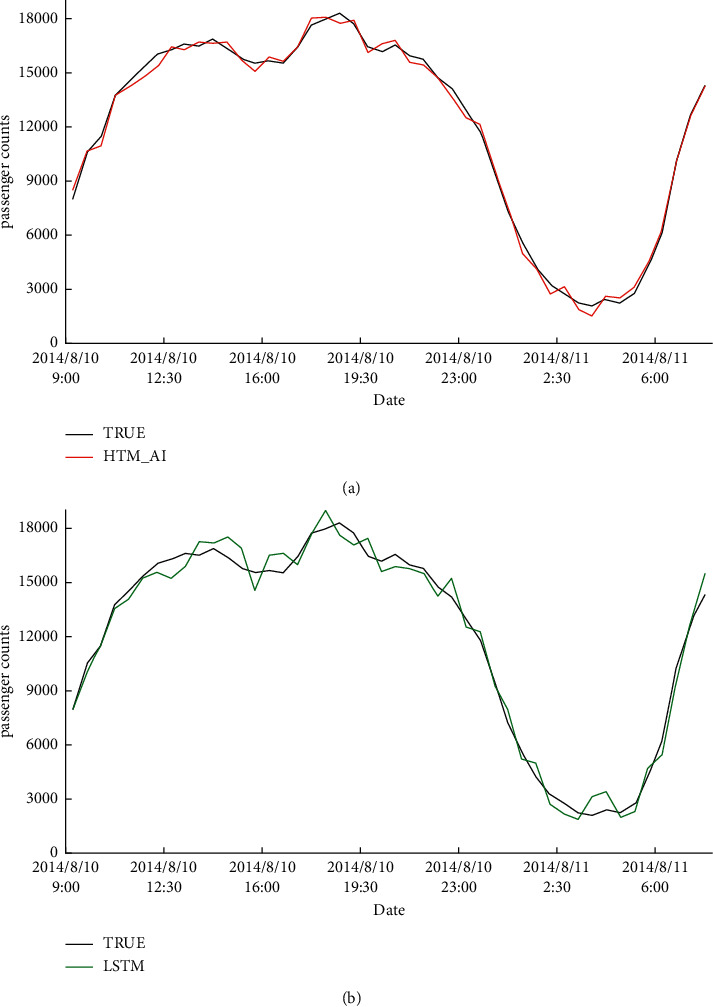
Predicted passenger counts. True data are shown in black. (a) The prediction results using HTM_AI, shown in red. (b) The prediction result using LSTM, shown in green.

Finally, we give the long-term prediction results on two datasets. Figures 13 and 14 show the transition of the prediction values by the proposed HTM_AI and the conventional HTM on NAB and NYC-Taxi, respectively. For each of the two algorithms, we plotted a representative result of one run with the closest total prediction error to the average among the 20 runs. Each figure also gives the true value in black that must be predicted. From the results, we can see HTM_AI can achieve a better accuracy than LSTM from a long-term interval. The prediction results of LSTM is not stable and derivate from the true data greatly. Even a decoder is employed to convert SDR to real value, the predicted output is still closer to the true data than LSTM. This indicates the proposed HTM has higher prediction accuracy, and the decoder can effectively generate the final output.

### 5.6. Results on Hyperparameter Tuning

The proposed self-adaptive HTM learning algorithm involves some extra hyperparameters besides the regular ones used in conventional HTM. The coefficients *α* and *ω* are most important for synaptic permanence update. In this section, we perform extensive hyperparameter tuning.

To evaluate their effects on the final performance, for *α*, which balances the two components in the definition of activation intensity, we chose five values: 0.1, 0.3, 0.5, 0.7 and 0.9. For *ω*, we follow the settings in the previous section.  Table 5 shows the prediction results with different configurations of *α* and *ω* on NAB and NYC-Taxi datasets, respectively.

From Table 5, we can see that when *α* is 0.1, the accuracy changes obviously with different values of *ω*. The highest accuracy is achieved when *ω* = 4.5. When *α* increases from 0.3 to 0.7, the accuracy of HTM_AI improves continuously, whereas it decreases when *α* reaches 0.9. This indicates that the two components of activation intensity should be balanced in order to get a better performance. So, we set *α* to 0.5 in the experiment. For *ω*, the accuracy under the different values varies on two datasets. On the NAB dataset, the best accuracy 98.3% is achieved when *α* = 0.5 and *ω* = 5.0, whereas on the NYC-taxi dataset, the best value 98.7% comes with *α* = 0.7 and *ω* = 4.0. The reason may be the statistic feature of the two datasets differs slightly. NYC-Taxi exhibits more cyclicity than NAB. However, if we choose *α* to be 0.5, the difference of accuracy under various *ω* is not obvious, no more than 0.5%.

## 6. Conclusions

To improve the representation capability of HTM and dynamically adjust the permanence value of synaptic connections, in this work, we proposed a novel HTM based on activation intensity. By introducing the activation intensity, more useful information about the input pattern is maintained on the HTM column. During learning the sequence, we improve the traditional Hebbian learning by presenting a column-sensitive self-adaptive TM algorithm, which employs a nonlinear updating strategy and adjusts the permanence according to the activation intensity of the column where the synapse locates. The experimental results on time-series input showed that the proposed HTM can better utilize the characteristics of input data. The prediction accuracy and error metric indicate our HTM achieved a higher accuracy than the conventional HTM and LSTM model. Furthermore, the time overhead revealed the proposed method can speed up HTM training by 29%–61%.

In future work, we will explore how to avoid the hyperparameter fine-tuning for various datasets and applications, and verify the effectiveness of the proposed method on other sequence learning tasks.

## Figures and Tables

**Algorithm 1:**
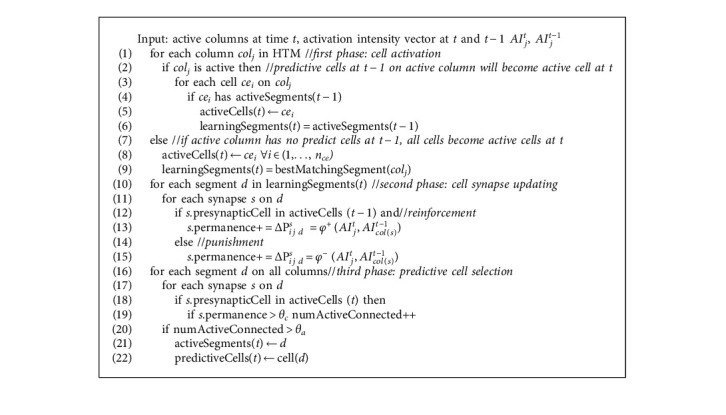
Temporal memory learning algorithm based on activation intensity.

**Table 1:** Model parameters for the proposed HTM and baseline HTM.

Parameter name	Value
Number of columns *N*	2048
Number of cells per column *M*	32
Number of active columns	40
Dendritic segment activation threshold	4
Initial synaptic permanence	0.2
Connection threshold for synaptic permanence	0.8
Synaptic permanence increment *ρ*^+^	0.1
Synaptic permanence decrement *ρ*^−^	0.1
Coefficient *α*	0.5

**Table 2:** Model parameters for the LSTM network.

Parameter name	Value
Number of hidden layer	32
Learning rate	0.001
Mini-batch	64

**Table 3:** Average accuracy (±standard derivation) with different numbers of neurons on NAB.

Configuration	Accuracy
HTM_AI-neurons (8)	0.978 (±0.0021)
HTM_AI-neurons (16)	0.976 (±0.0025)
HTM_AI-neurons (24)	0.977 (±0.0023)
HTM_AI-neurons (32)	0.978 (±0.0022)
HTM-neurons (8)	0.963 (±0.0013)
HTM-neurons (16)	0.962 (±0.0019)
HTM-neurons (24)	0.963 (±0.0021)
HTM-neurons (32)	0.964 (±0.0017)

**Table 4:** Average accuracy (±standard derivation) with different numbers of neurons on NYC-Taxi.

Configuration	Accuracy
HTM_AI-neurons (8)	0.983 (±0.0031)
HTM_AI-neurons (16)	0.984 (±0.0033)
HTM_AI-neurons (24)	0.981 (±0.003)
HTM_AI-neurons (32)	0.982 (±0.0028)
HTM-neurons (8)	0.971 (±0.0020)
HTM-neurons (16)	0.97 (±0.0017)
HTM-neurons (24)	0.969 (±0.0018)
HTM-neurons (32)	0.972 (±0.0021)

**Table 5:** Prediction accuracy of different configurations on hyperparameter.

		Accuracy (%)	
*α*	*ω*	NAB	NYC-Taxi
0.1	3.5	89.5	91.7
	4.0	90.1	92.5
	4.5	93.1	93.8
	5.0	92.9	92.0

0.3	3.5	96.3	96.2
	4.0	95.1	94.1
	4.5	94.7	96.7
	5.0	95.9	95.9

0.5	3.5	97.8	97.9
	4.0	98.0	98.1
	4.5	98.1	97.7
	5.0	98.3	98.4

0.7	3.5	98.0	96.8
	4.0	97.3	98.7
	4.5	96.7	97.7
	5.0	98.1	96.8

0.9	3.5	94.2	94.1
	4.0	95.8	93.8
	4.5	94.6	92.6
	5.0	95.3	93.3

## Data Availability

The Numenta Anomaly Benchmark (NAB) dataset used to support the findings of this study is available at https://github.com/numenta/NAB. The NYC-Taxi dataset used to support the findings of this study is available at http://www.nyc.gov/html/tlc/html/about/trip_record_data.shtml.
